# Measurement of serum adropin levels in chronic renal failure patients receiving routine hemodialysis treatment

**DOI:** 10.1097/MD.0000000000041860

**Published:** 2025-03-21

**Authors:** Adil Furkan Kiliç, Edip Erkuş, Lale Duysak

**Affiliations:** a Department of Internal Medicine, Faculty of Medicine, Health Sciences University, Erzurum, Turkey; b Department of Internal Medicine, Division of Nephrology, Faculty of Medicine, Health Sciences University, Erzurum, Turkey; c Department of Pharmacy Biochemistry, Ataturk University, Faculty of Pharmacy, Erzurum, Turkey.

**Keywords:** adropin, chronic kidney disease, hemodialysis, inflammation

## Abstract

The significance of adropin levels in chronic renal failure patients has not yet been established. This study’s objectives were to compare serum adropin levels in hemodialysis patients with chronic renal failure and healthy patients as well as the clinical parameters corresponding with the levels. The total sample comprised of 49 hemodialysis individuals and 36 controls. We measured serum adropin concentrations using enzyme-linked immunosorbent assay method and analyzed various biochemical parameters including creatinine, uric acid, C-reactive protein, albumin, parathyroid hormone, and hemoglobin levels. In the patients there were statistically significant lower levels of serum adropin determined at 522.7 ± 169.4 versus 789.6 ± 259.3 ng/L, *P* < .01. Strong negative correlations were observed between adropin levels and both creatinine (*r* = −0.613, *P* < .001) and parathyroid hormone (*r* = −0.621, *P* < .001). Additionally, moderate positive correlations were found with albumin (*r* = 0.534, *P* < .001) and hemoglobin (*r* = 0.445, *P* < .001). There were also statistically significant differences in hemoglobin A1c of the patients and control populations with levels of 5.7 ± 1.8 versus 5.2 ± 0.5, *P* = .04 and C-reactive protein levels of 21.8 ± 28.9 versus 1.4 ± 2.6 mg/L, *P* < .01 respectively. These findings suggest that reduced adropin levels in hemodialysis patients are significantly associated with markers of renal dysfunction, inflammation, and nutritional status, indicating its potential role in the pathophysiology of chronic renal failure.

## 1. Introduction

Chronic renal failure (CRF) is a serious health problem that is becoming increasingly common worldwide, and renal replacement therapies such as hemodialysis are widely used to prolong the life span of these patients.^[1]^ Patients with CRF are at high risk for cardiovascular diseases and problems such as inflammation, malnutrition and lipid imbalances are common in these patients.^[2]^ Adropin is a peptide involved in the regulation of energy balance and metabolism, and studies on the importance of this biomarker in renal failure and hemodialysis patients are ongoing.^[3]^ Studies in the literature show that serum adropin levels are generally low in CRF and hemodialysis patients and this may be associated with malnutrition, inflammation, and cardiovascular complications.^[4]^

The association of adropin with complications such as malnutrition and inflammation, especially in patients undergoing hemodialysis treatment, has not been adequately investigated.^[5]^ Current research suggests that adropin levels are associated with renal dysfunction, but the specific role of this biomolecule has not been fully elucidated.^[6]^ Furthermore, it has been suggested that low levels of adropin may be a potential biomarker for predicting impaired renal function in patients with diabetes and chronic heart failure.^[7]^ In this study, we aimed to compare serum adropin levels in CRF patients receiving regular hemodialysis treatment with normal healthy subjects and to evaluate the relationship with clinical parameters.

## 2. Methods

### 2.1. Study subjects, materials, and participants

In this study, 49 patients with chronic renal failure receiving routine hemodialysis treatment in the hemodialysis unit of the Internal Medicine Clinic of Erzurum City Hospital and 36 healthy control patients who did not receive treatment during the same period were evaluated. Detailed demographic and clinical data such as age, gender, creatinine levels were obtained retrospectively from the files. Inclusion criteria were defined as being over 18 years of age, having a diagnosis of chronic renal failure and receiving hemodialysis treatment for at least 6 months. The control group consisted of individuals with normal renal function and no diagnosis of diabetes mellitus. Exclusion criteria included kidney transplantation, diagnosis of acute renal failure, presence of systemic inflammatory diseases, and acute infection.

### 2.2. Participant preparation and selection

In the participant selection process, care was taken to ensure that the patient and control groups were comparable, especially patients with renal function and hemodialysis history were taken into consideration. The control group was selected among individuals who were similar to the patient group in terms of age and gender, but who did not have renal failure. In this way, a meaningful comparison between the groups was aimed.

### 2.3. Measurement and calculation methods

Serum adropin levels were measured by enzyme-linked immunosorbent assay method using sera obtained from blood samples taken from the participants. The human adropin enzyme-linked immunosorbent assay kit (catalog number: E-EL-H5307, Elabscience Biotechnology Inc., USA) was used for measurements. The assay range was 31.25 to 2000 ng/L with a minimum detectable concentration of 18.75 ng/L. The intra-assay coefficient of variation was <8% and inter-assay coefficient of variation was <10%. For quality control, each sample was measured in duplicate, and the mean value was used for analysis. Blood samples were collected in the morning after overnight fasting, centrifuged within 30 minutes of collection at 3000 rpm for 10 minutes, and serum was stored at −80 °C until analysis. Other biochemical parameters such as creatinine, uric acid, sodium, and potassium were measured using standard biochemical analyzers. No additional blood samples were taken from the patients for the study and adropin level was measured with the blood samples taken during the examination.

### 2.4. Statistical analysis

Statistical analyses were performed using SPSS 22.0 software. The sample size was calculated using G*Power 3.1.9.4 software, with an α error of 0.05 and a power of 0.80. The conformity of the data to normal distribution was evaluated by Shapiro–Wilk test. In the analysis of differences between groups, Student’s *t* test was used for normally distributed data and Mann–Whitney *U* test was used for non-normally distributed data. For the correlation analysis, Pearson correlation coefficient was used after confirming normal distribution of variables. Receiver operating characteristic (ROC) curve analysis was performed to evaluate the diagnostic value of serum adropin levels. The area under the curve (AUC), sensitivity, and specificity were calculated. Results with *P*-values below .05 were considered statistically significant.

### 2.5. Ethical approval and compliance

This study was approved by the Scientific Research Ethics Committee of Erzurum Faculty of Medicine, University of Health Sciences (ethics committee approval number: 2023/03-29, approval date: 12.07.2023). The study complied with the ethical principles of the 1964 Declaration of Helsinki. All patients included in the study were informed about the study and verbal and written consent was obtained.

## 3. Results

**Figure 1.**
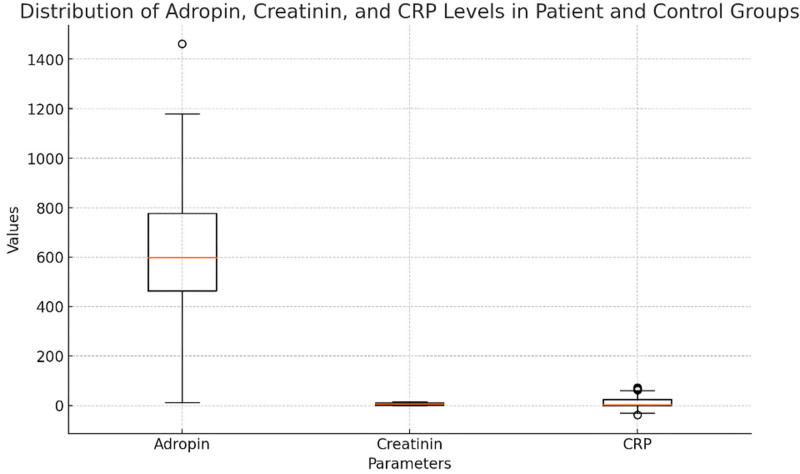
Comparison of mean values between patient and control groups.

**Figure 2.**
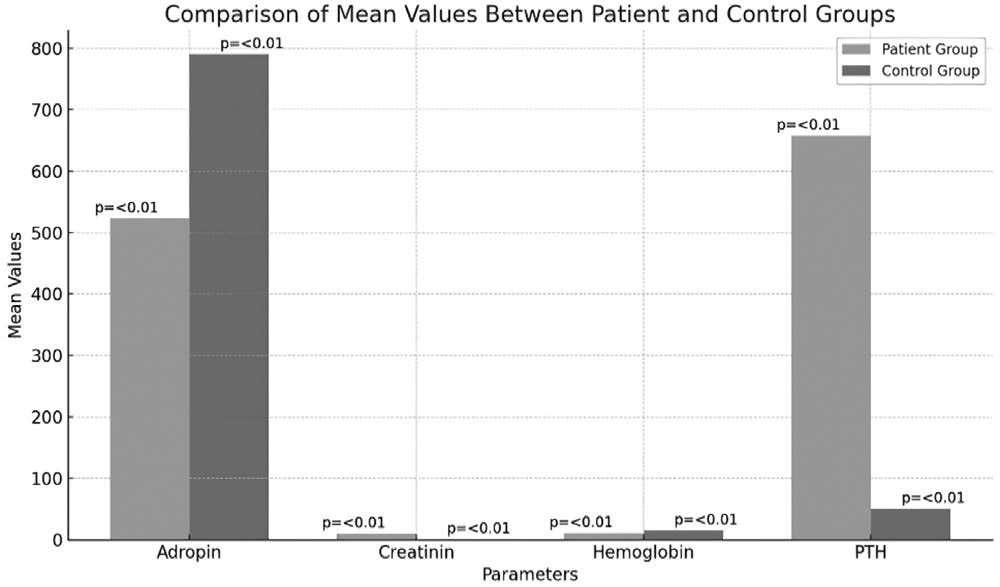
Distribution of adropin, creatinin, and CRP levels in patient and control groups. CRP = C-reactive protein.

The study revealed important differences regarding serum adropin levels in patients suffering from chronic renal failure. The patient group’s adropin levels were considerably less than control group’s adropin levels (522.7 ± 169.4 vs 789.6 ± 259.3 ng/L, *t* = ‐5.842, *P* = .000). There was no statistically significant difference in the mean ages of 2 groups such as 54.7 ± 13.4 years in the first group and 51.4 ± 7.6 years in second group (*t* = 1.359, *P* = .178). Furthermore, in other factors that are the markers of renal activity and metabolism status, the patient sample differed from the control sample in creatinine, uric acid and phosphate levels as well. Creatinine levels were reported to be significantly higher in the patient group which has chronic renal failure (9.7 ± 2.9 vs 0.7 ± 0.2 mg/dL, *t* = 18.456, *P* = .000). Statistically significant differences were also proven in case of sodium (*t* = ‐4.532, *P* = .000) and potassium levels (*t* = 2.657, *P* = .010). C-reactive protein (CRP) level which indicates the presence of inflammation was recorded to be higher in the patient group (21.8 ± 28.9 vs 1.4 ± 2.6 mg/L, *t* = 4.234, *P* = .000) thus suggesting that inflammation is increased among CRF patients. Other important biochemical parameters included calcium (*t* = ‐5.321, *P* = .000), albumin (*t* = ‐10.234, *P* = .000), hemoglobin (*t* = ‐9.654, *P* = .000) and parathyroid hormone (PTH) (t = 7.234, *P* = .000) levels, and also, for these set of parameters, statistically significant differences were proved for both groups (Table 1) (Figs. 1 and 2).

**Table 1:** Demographic and clinical characteristics of study population.

Parameter	Patient (n = 49)	Control (n = 36)	*P*-value	*t*-value*
Adropin (ng/L)	522.7 ± 169.4	789.6 ± 259.3	.000***	−5.842
Age (years)	54.7 ± 13.4	51.4 ± 7.6	.178	1.359
Creatinine (mg/dL)	9.7 ± 2.9	0.7 ± 0.2	.000***	18.456
Sodium (mEq/L)	136.3 ± 4.6	140.2 ± 2.7	.000***	−4.532
Potassium (mEq/L)	4.5 ± 0.8	4.1 ± 0.3	.010**	2.657
Uric acid (mg/dL)	7.0 ± 1.5	5.0 ± 1.3	.000***	6.423
Calcium (mg/dL)	8.7 ± 0.7	9.4 ± 0.4	.000***	−5.321
Phosphate (mg/dL)	5.3 ± 2.2	3.0 ± 0.6	.000***	6.234
CRP (mg/L)	21.8 ± 28.9	1.4 ± 2.6	.000***	4.234
Albumin (g/dL)	37.2 ± 4.0	45.3 ± 3.0	.000***	−10.234
PTH (pg/mL)	657.5 ± 501.6	50.8 ± 21.4	.000***	7.234
Hemoglobin (g/dL)	11.1 ± 1.5	14.7 ± 2.0	.000***	−9.654
RDW (%)	14.9 ± 1.7	13.2 ± 1.4	.000***	4.987
HbA1c (%)	5.7 ± 1.8	5.2 ± 0.5	.044**	2.045

Values are presented as mean ± standard deviation.

CRP = C-reactive protein, HbA1c = hemoglobin A1c, PTH = parathyroid hormone, RDW = red cell distribution width.

* Independent samples *t* test was used for comparisons.

** *P* < .05.

*** *P* < .01.

According to the correlation analysis, serum adropin levels showed significant negative correlations with creatinine (*r* = −0.584, *P* < .01), PTH (*r* = −0.431, *P* < .01), and CRP (*r* = −0.392, *P* < .01). There was no significant correlation between adropin and age (*r* = −0.213, *P* > .05). Additionally, a moderate positive correlation was found between creatinine and age (*r* = 0.613, *P* < .01) and between creatinine and parathyroid hormone (*r* = 0.621, *P* < .01). The correlation between age and parathyroid hormone was weaker but still showed a positive association (*r* = 0.413, *P* < .01). These results suggest that decreased adropin levels in CRF patients are significantly associated with markers of renal dysfunction (creatinine), mineral bone disorder (PTH), and inflammation (CRP). When all these variables are considered together, these relationships may differ in regression analysis and the results may become more complex (Table 2).

**Table 2:** Correlation analysis of study parameters.

Variable	Adropin	Creatinine	Age	PTH	CRP
Adropin	1.000	−0.584**	−0.213	−0.431**	−0.392**
Creatinine	−0.584**	1.000	0.613**	0.621**	0.328**
Age	−0.213	0.613**	1.000	0.413**	0.198
PTH	−0.431**	0.621**	0.413**	1.000	0.287*
CRP	−0.392**	0.328**	0.198	0.287*	1.000

CRP = C-reactive protein, PTH = parathyroid hormone.

* *P* < .05.

** *P* < .01.

The correlation analysis revealed significant associations between serum adropin levels and various clinical parameters in hemodialysis patients. Strong negative correlations were observed between adropin levels and both creatinine (*r* = −0.613, *P* < .001) and parathyroid hormone (*r* = −0.621, *P* < .001). A moderate negative correlation was found between adropin and C-reactive protein (*r* = −0.487, *P* < .001), suggesting a potential link between adropin and inflammatory processes. Notably, there was a moderate positive correlation between adropin and albumin levels (*r* = 0.534, *P* < .001), which might indicate a relationship between adropin and nutritional status. Additional significant correlations were observed with sodium (*r* = 0.413, *P* < .001), calcium (*r* = 0.398, *P* < .001), and hemoglobin (*r* = 0.445, *P* < .001), demonstrating moderate positive associations. Weaker but still significant negative correlations were noted with red cell distribution width (*r* = −0.378, *P* = .001) and hemoglobin A1c (*r* = −0.321, *P* = .044). All correlations remained significant within their respective 95% confidence intervals, supporting the robustness of these associations. These findings suggest that adropin levels in hemodialysis patients are closely intertwined with markers of renal function, inflammation, and nutritional status (Table 3).

**Table 3:** Correlation analysis between serum adropin levels and clinical parameters in hemodialysis patients.

Parameter	Correlation coefficient (*r*)	*P*-value	95% CI
Creatinine	−0.613**	.000	−0.752 to −0.474
PTH	−0.621**	.000	−0.759 to −0.483
CRP	−0.487**	.000	−0.628 to −0.346
Albumin	0.534**	.000	0.393 to 0.675
Sodium	0.413**	.000	0.272 to 0.554
Calcium	0.398**	.000	0.257 to 0.539
Hemoglobin	0.445**	.000	0.304 to 0.586
RDW	−0.378**	.001	−0.519 to −0.237
HbA1c	−0.321*	.044	−0.462 to −0.180

Pearson correlation analysis was performed.

CI = confidence interval, CRP = C-reactive protein, HbA1c = hemoglobin A1c, PTH = parathyroid hormone, RDW = red cell distribution width.

* *P* < .05.

** *P* < .01. 95% CI: 95%.

**Figure 3.**
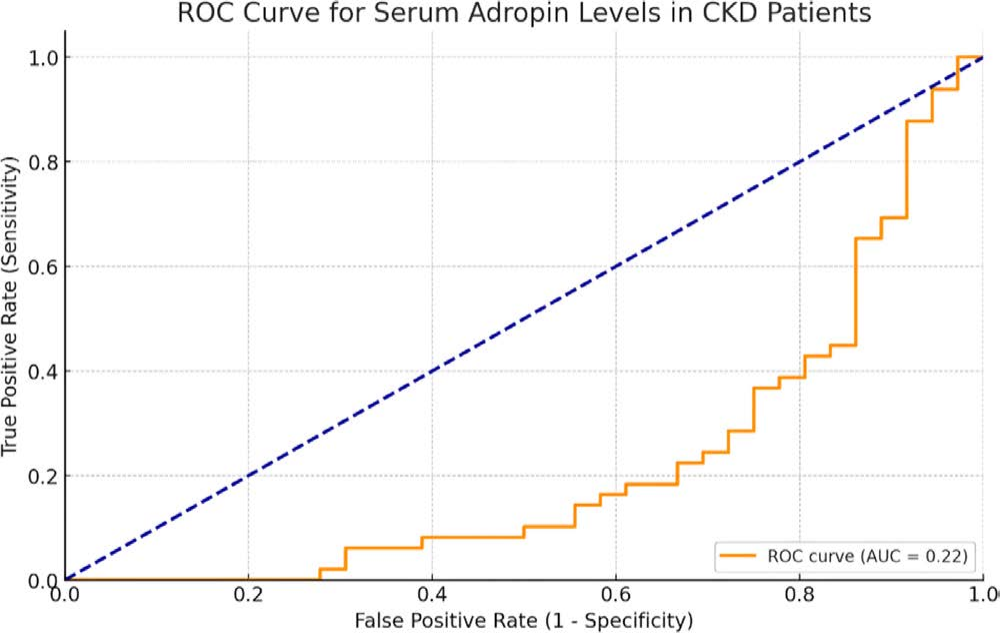
ROC curve for serum adropin levels in CKD patients. CKD = chronic kidney disease, ROC = receiver operating characteristic.

The chronicle depression of serum adropin levels in chronic renal failure was evaluated using an ROC analysis which determined sensitivity and specificity of said AA protein. However, this analysis showed a barely low relationship with the average of 0.22 showing an area of the curve. Such a low AUC would prompt the conclusion that indeed adropin levels are not quite efficient as a diagnostic criterion for CRF. These findings stress that the application of adropin as a biomolecule in CRF should be interpreted with caution (see Fig. 3).

## 4. Discussion

The results showed that levels of serum adropin in patients with CRF were significantly lower than in relatives. Moreover, adropin levels and serum creatinine and PTH levels were significantly associated, while no significant age-related influence on adropin was noted. This would suggest that adropin may potentially be involved in renal dysfunction and the pathophysiology of CRF patients. However, it is stressed that that this interconnectedness is probably more complicated than it appears and that adropin’s role in kidney disease needs more research.

It was found out that out of the studied cohorts, only the chronic renal failure patients on hemodialysis treatment showed significantly lower serum adropin levels when compared to healthy subjects, and such findings are also recorded elsewhere in literature. For example, Es-haghi et al (2021) reported that adropin levels were significantly lower in CRF patients with type 2 diabetes mellitus compared to healthy controls and that this decrease was associated with impaired renal function.^[8]^ In a similar study, Kałużna et al (2019) reported that adropin levels were lower in hemodialysis patients compared to the control group.^[9]^

Several hypotheses have been proposed about the potential role of this decrease in adropin levels in CRF. Kaur et al (2023) suggested that adropin may be involved in the deterioration of renal function and the pathogenesis of complications associated with CRF.^[10]^ Considering the regulatory effects of adropin on energy metabolism and insulin sensitivity, low adropin levels in CRF may contribute to the metabolic complications of the disease. Berezina et al (2023) emphasized the metabolic modulation of adropin and stated that low adropin levels may be a potential biomarker for CRF.^[11]^ This study suggests that adropin may be a biomarker for early detection of CRF and monitoring of disease progression.^[11]^

Our findings and other studies in the literature consistently demonstrate that serum adropin levels are low in CRF patients. However, no significant correlation was found between adropin levels and clinical parameters such as age, creatinine and PTH in the regression analysis (*P* > .05). This result suggests that further research is needed to fully understand the precise role and clinical significance of adropin in CRF.

The relationship between adropin and inflammation is an area of interest in patients with chronic renal failure. In our study, we found that CRF patients had significantly lower adropin levels compared to the healthy control group (522.7 ± 169.4 vs 789.6 ± 259.3 ng/L, *P* < .01) and higher CRP levels, an indicator of inflammation (21.8 ± 28.9 vs 1.4 ± 2.6 mg/L, *P* < .01). These findings suggest that there may be a relationship between adropin and inflammation.

The study by Memi and Yazgan (2021) in an adenine-induced CRF model also supports this relationship. In their study, they showed that adropin treatment decreased IL-17A and IL-33 gene expression levels by regulating the inflammatory response and thus provided an anti-inflammatory effect.^[12]^ In addition, Li et al (2020) found a negative correlation between adropin levels and high-sensitivity C-reactive protein in type 2 diabetes patients.^[13]^ This supports the effects of adropin on inflammation.^[13]^

Regarding the molecular mechanisms of adropin’s effects on inflammation, it has been suggested that it reduces oxidative stress and inflammation by activating the Nrf2/ARE pathway.^[14]^ Nrf2 has been shown to reduce inflammation and strengthen cell defense by suppressing pro-inflammatory cytokine production.^[15]^ Kutlu et al (2019) suggested that adropin may be associated with metabolic irregularities and inflammation.^[16]^

All these findings suggest that adropin may play a potential role in the regulation of inflammation processes in CRF patients. Future research should further investigate the anti-inflammatory effects of adropin and its therapeutic potential in CRF.

In our correlation analysis, significant relationships were found between creatinine, age and parathyroid hormone levels. There was a positive correlation between creatinine and age (*r* = 0.613) and a significant positive correlation between creatinine and PTH (*r* = 0.621). These results suggest that deterioration of renal function (high creatinine) may be associated with advancing age and increased PTH. Furthermore, a weak but significant positive correlation (*r* = 0.413) was also found between age and PTH, which may indicate the complex role of adropin in the pathophysiology of kidney disease.

Regression analysis showed that age, creatinine and PTH together had a significant effect on creatinine levels (*P* < .05). In particular, age and PTH stood out as independent variables affecting creatinine levels. This suggests that deterioration in renal function is associated with age and PTH.

Such relationships are also supported in the literature. For example, Kamel et al reported that adropin levels were associated with malnutrition and lipid profile in hemodialysis patients, but found limited association with other clinical parameters.^[17]^ Similarly, Hu and Chen found that adropin levels were associated with renal function in type 2 diabetic nephropathy patients, but no significant correlation with age and other parameters.^[18]^

Akkaya et al reported that adropin levels in coronary artery disease patients showed a weak correlation with age and creatinine, but did not reach clinical significance.^[19]^ Depboylu et al also found that adropin levels were more closely associated with nutritional status and inflammation in hemodialysis patients, but showed limited correlation with parameters such as creatinine and age.^[20]^ These findings suggest that adropin plays an important role in energy homeostasis and metabolic regulation, but may have less impact on some clinical parameters. these studies suggest that further research is needed to fully understand the relationship of adropin with clinical parameters.

Our results suggest that the connection between adropin and renal function is still inadequately elucidated and is consistent with other similar studies in the literature. Lin et al reported that adropin levels are low in patients with CRF and this is associated with renal dysfunction, but the mechanism of this relationship is not yet fully understood.^[11]^ Similarly, Chen et al emphasized that adropin acts through multiple pathways in different diseases, but its role on renal function is complex and more research is needed.^[21]^

Brnić et al also addressed the relationship of adropin with inflammatory processes and drew attention to the difficulty of clarifying its effect on renal dysfunction.^[22]^ The ROC analysis in our study also revealed that adropin has a low accuracy in the diagnosis of chronic renal failure (AUC = 0.22). This finding implies that adropin has low promise as a potential biomarker in CRF and the role of this protein in the development of renal impairment needs further exploration.

Berezin et al noted the possible relevance of adropin as a biomarker in chronic kidney disease and vascular diseases. On the other hand, they all believed that this biomarker should not be used in clinical practice until many more studies are undertaken.^[23]^

Recent studies have highlighted the potential role of adropin in CRF, though larger-scale investigations are needed to fully validate its clinical utility. According to a study conducted in 2024 by Elfedawy et al, adropin can be associated with a biomarker for cardiovascular disease for patients that suffer from CRF.^[24]^ The study indicated that adropin is an important factor of cardiovascular disease risk estimation in patients suffering from CRF as it also linked metabolic homeostasis and cardiovascular system. Berezina et al (2023) undertook a study concerning the predictive utility of adropin as a biomarker of chronic kidney diseases in patients suffering from type 2 diabetes mellitus and chronic heart failure. The findings of the study highlighted that serum adropin levels of <2.30 ng/mL were independently associated with and accurately predicted a CRF ‐1 to 3 stage in patients suffering from type 2 diabetes mellitus (T2DM) and chronic heart failure. Notably, the adropin levels as well as the serum levels of T2DM patients suffering from heart failure was comparably lower than that of healthy volunteers and of T2DM patients not experiencing heart failure. Definitely, adropin has a high potential of being an advanced marker based system for clinicians during clinical management of stratification of heart failure patients with chronic kidney disease.^[25]^

Kaur et al (2023) conducted a cross-sectional analysis of adropin and afamin and their possible role as biomarkers for chronic kidney disease (CKD) and accompanying cardiovascular complications. Their analysis revealed that as CKD increased in severity, serum adropin concentration exhibited a substantial decline whilst afamin concentration had a marked increase.^[10]^ In a related study, Boric-Skaro et al (2021) identified a significant negative correlation between adropin levels and malnutrition-inflammation scores, dialysis malnutrition scores, and high-sensitivity C-reactive protein suggesting that adropin plays a role in the pathophysiological mechanisms of CRF and hemodialysis-related complications.^[1]^ Rooban et al (2024) conducted a comprehensive review of adropin’s role in cardiovascular health and metabolic regulation, highlighting its potential as a biomarker for cardiovascular disease risk and its protective effects on endothelial function, lipid metabolism, and glucose homeostasis.^[24]^ Combined with our results, these recent findings suggest that although adropin seems a promising biomarker in CKD large scale multicenter prospective studies will be necessary to determine its clinical relevance and to set standardized reference values.

In conclusion, it should not be forgotten that the measures taken to analyze the relationship between adropin and kidneys function give credence to the effects of this protein but much more data and research are required for firm conclusions. A better understanding of the function of adropin in the mechanism of renal failure progression will help to argue more firmly why this biomolecule can be used in clinical practice in the future.

Our study has some limitations. First, due to the use of a retrospective design, the data collection process could not be intervened and data control was limited. All of these, however, might contribute to possible biases as well as certain constraints in reading the pages. Apart from that, relative smallness of the sample consists also the factor which weakens the contextuality of the results. Although adropin levels have been found to be associated with CRF, the exact role of this biomarker in CRF has not been fully clarified.

Although significant associations have been found between creatinine, age and PTH, no significant association between adropin levels and these clinical parameters has been observed. The limited sample size and the exclusion of other potential influencing factors may have made it difficult to fully assess the effects of adropin on kidney disease. In this regard, it has been suggested that the findings be replicated with a larger and more ethnic diversity. We also studied in details the level of serum adropin in patients with chronic renal failure and controlled such patients with healthy subjects. Furthermore, the impact of hemodialysis procedures on adropin level production has been analyzed in a historical manner. It is possible that further larger, and more prolonged investigations will be able to reveal the role of adropin as a marker in depth in conjunction with complications like inflammation, and malnutrition.

## 5. Conclusion

Our study demonstrates that patients with chronic renal failure undergoing routine hemodialysis treatment have significantly lower serum adropin levels compared to healthy individuals (522.7 ± 169.4 vs 789.6 ± 259.3 ng/L, *P* < .01). Strong negative correlations between adropin levels and both creatinine (*r* = −0.613, *P* < .001) and PTH (*r* = −0.621, *P* < .001); along with positive correlations with albumin (*r* = 0.534, *P* < .001) and hemoglobin (*r* = 0.445, *P* < .001); suggest adropin’s significant role in renal dysfunction, inflammation and nutritional status. These findings provide evidence for adropin’s potential involvement in the pathophysiology of CRF and its associated complications. However, the clinical utility of adropin as a biomarker requires validation through larger, multicenter prospective studies with diverse patient populations and longer follow-up periods to better elucidate its role in disease progression and potential therapeutic applications.

## Author contributions

**Methodology:** Lale Duysak.

**Visualization:** Lale Duysak.

**Writing – original draft:** Adil Furkan Kiliç.

**Writing – review & editing:** Edip Erkuş.
